# Paediatric care in hospitals in Kyrgyzstan and Tajikistan: impact of a quality improvement initiative

**DOI:** 10.7189/jogh.15.04272

**Published:** 2025-10-24

**Authors:** Bayan Babayeva, Zaure Ospanova, Obidjon Aminov, Venera Shukurova, Nurshaim Tilenbaeva, Shoira Yusupova, Alexandre Avaliani, Martin W Weber, Sophie Jullien, Aigul Kuttumuratova, the Kyrgyzstan-Tajikistan Quality of Hospital Care for Children study group, Venera Shukurova, Aizat Asanova, Begaim Akelbaeva, Aitbubu Abdyrahmanova, Farida Faizova, Nazira Shermatova, Gulnaz Majitova, Rakhmatullo Shobonov, Jurabek Ishmerzoev, Shukhrat Zubaydzoda, Iftikhor Mirzoda, DIB Dushanbe, Shamsia Narzulloyeva, Ekaterina Stasii, Marina Taratina, Dmitry Yasakov, Elena Kulik

**Affiliations:** 1 National Centre of Medical Education, Astana Medical University, Astana, Kazakhstan; 2 Department of Child and Adolescent Health, Ministry of Health and Social Protection of Population, Dushanbe, Tajikistan; 3 Pediatric Department, Kyrgyz State Medical Institute of Continuous Education, Bishkek, Kyrgyzstan; 4 Office for Quality of Care and Patient Safety, World Health Organization Regional Office for Europe, Athens, Greece; 5 World Health Organization Country Office in Tajikistan, Dushanbe, Tajikistan; 6 School of Social Sciences, Georgian Institute of Public Affairs, Tbilisi, Georgia; 7 Division of Country Health Policies and Systems, Policy and Governance in Health Unit, World Health Organization Regional Office for Europe, Copenhagen, Denmark; 8 World Health Organization European Centre for Primary Health Care, World Health Organization Regional office for Europe, Almaty, Kazakhstan

## Abstract

**Background:**

Adequate quality of paediatric care remains one of the key factors for reducing child mortality and improving child health in Central Asia. We aimed to assess the quality of paediatric care in hospitals in Kyrgyzstan and Tajikistan before and after a two-year intervention. We implemented a multifaceted approach to improve healthcare quality, focussing on case management, hospital policies, and service organisation.

**Methods:**

We assessed the quality of care in nine hospitals in Kyrgyzstan and ten hospitals in Tajikistan, using a World Health Organization paediatric hospital care assessment tool. The assessment considered three main pillars of care: hospital support services, case management, and policies and organisation of services. We collected the data through direct observation, analysis of general hospital data, case files, policies and procedures, clinical guidelines, reports and interviews with staff, management, and caretakers. We compared the scores (0–3) at baseline with those at the end of the project.

**Results:**

We found many areas of inadequate care at the baseline assessments in both countries. The endline assessment showed improvements in areas such as clinical management of acute respiratory infections, staff training, hospital policies, and the reduction of unnecessary painful procedures. The management of pneumonia, including the use of appropriate antibiotics and oxygen therapy, has improved in most hospitals in both countries. The mean score for assessing suspected pneumonia improved from 2.1 to 2.7 (*P* = 0.018) in Kyrgyzstan and from 1.6 to 2.1 (*P* = 0.020) in Tajikistan. Unnecessary, painful, and invasive procedures decreased from 70% to 48% of cases in Tajikistan and from 66% to 36% in Kyrgyzstan. Gaps remained in infrastructure, chronic disease management, and the rational use of medicines.

**Conclusions:**

A set of quality improvement measures led to improvements in the hospitals. Irrational antibiotic prescribing, overmedicalisation, and unjustified hospitalisation continue to be issues related to the broader health system. Systemic issues, including human resource constraints, infrastructure limitations, and supply shortages, need to be addressed. Scaling up the project to other hospitals would improve the overall quality of care.

Adequate quality of paediatric care remains one of the key factors for reducing infant and child mortality and improving child health. Poor-quality hospital care is a global challenge, and the World Health Organization (WHO) European Region is not an exception. A child born in the countries of Central Asia is three times as likely to die before the age of 5 years as a child born in a European Union country [24].

Over the last two decades, evidence has shown a substantial scope for improving the quality of childcare, as indicated by published and unpublished assessments in district-level hospitals across various countries [1–5]. Applied health service assessments from both countries revealed suboptimal quality of care (QoC) in the treatment of common childhood conditions [6,7].

To address these issues, we implemented a project on quality improvement (QI) of hospital care in Kyrgyzstan and Tajikistan from 2021 to 2024 to reduce maternal, newborn, and child deaths. The project was initiated to strengthen the national and local health system’s capacity to accelerate ending preventable child mortality by improving the QoC. Both countries perceived the baseline and endline measurements of QoC in project hospitals as an evidence-based generation of knowledge on the effectiveness and feasibility of the implemented multifaceted approaches for improving QoC, which can then be scaled up across other hospitals. These activities were aligned with the development of the national governance in QoC and local QI committees.

## METHODS

### The project interventions

The project followed the QI approach in the WHO European Framework for improving reproductive, maternal, newborn, and child QoC, which describes a systematic process for improving QoC [8]. Within the two years following the baseline assessment, multiple interventions aimed at improving QoC were implemented in both countries. Briefly, this integrated approach included a review of existing policies and clinical guidelines, identifying needs in their update, revision, and harmonisation, national capacity-building workshops on the use of updated national guidelines based on the WHO Pocket book of hospital care for children [9], training on QI methods, supportive supervision, as well as semi-annual collaborative improvement meetings with participation of all project hospitals. In a consultative process, the hospitals developed individual QI plans based on the baseline assessment results. More details of the project activities and interventions in Kyrgyzstan and Tajikistan are described in the country-specific paper published in this series [10], as well as in two parallel papers [11,12] that address related aspects of maternal and neonatal care.

### Project hospitals

In both countries, the respective Ministry of Health, in coordination with WHO, selected the project hospitals, aiming to get a representative selection of hospitals in the country, purposefully including neglected regions and areas. Teams of international and national experts conducted assessments in nine selected hospitals in Kyrgyzstan, with a baseline in October 2021 and an endline in October 2023, and in ten hospitals in Tajikistan, with a baseline in November 2021 and an endline in November 2023.

### Assessment tool and methods

We conducted an assessment of the quality of hospital care for children using an adapted WHO paediatric assessment tool [13]. The tool evaluated the availability and appropriate use of resources, case management, and key hospital policies, focussing on health system issues. The tool assesses 13 different aspects of child healthcare from admission to discharge and follow-up. Overall, the assessment tool considered three main pillars: hospital support services, case management, and policies and organisation of services. We collected the data through direct observation, analysis of medical records, policies and procedures, clinical guidelines, inventory and operational reports, as well as interviews with staff, management, and caregivers of sick children. We applied a scoring system of 0–3, which we then visualised through colour coding in heat charts. We assessed each area based on information from different sources to get the overall score.

A score of ‘3′ indicated care corresponding to international standards (no need for improvement or need for minor improvements only); ‘2’ indicated substandard care but no significant hazard to health or violation of human rights (need for some improvement to reach standard care);‘1’ indicated inadequate care with serious health hazards or violation of children’s rights (*e.g.* omission of evidence-based interventions or information with consequent risk for health or violation of human rights), indicating a need for substantial improvement to reach standard care; and ‘0’ indicated very poor care with consequent systematic and severe hazards to the health of children.

We divided each area into sub-areas with several variables contributing to each. We calculated the score for each area and sub-area as the mean (x̄) of the scores for all included variables. We undertook a chart review of a minimum of 30–35 cases in each facility, randomly chosen among children aged six months to five years admitted in the previous 1–3 months with acute respiratory infections (ARIs), diarrhoea, or fever. The WHO assessment tool recommends a sample size of 30–35 cases. We did not stratify the diagnosis since most cases were ARIs with fever. We analysed the nine QoC indicators, including diagnostics, treatment, painful procedures, and hospitalisation.

The assessors met after the assessment in the hospital visit to discuss findings, attribute scores, and prepare for a feedback meeting. Hospital managers and staff discussed the findings and suggested actions at the summary meetings. After all assessments, we presented the consolidated findings to the Ministries of Health and key partners. More details on the methods and country-level results are described in the country-specific paper [10].

### Assessment teams

Three teams of national and international experts conducted the assessments. All national assessors were trained in a two-day assessment workshop. Each team consisted of at least two paediatricians and one medical nurse, led by an international expert. To ensure consistency of methods and scoring, the international and national experts were the same throughout both assessments.

### Comparison and data management

The assessment teams summarised the x̄ values for each assessment area and sub-area. We transcribed the data into an Excel Sheet with coded facilities and calculated the differences in scores between the baseline and endline assessments. Additionally, we visualised the changes between baseline and endline assessment scores with colour coding as follows: a change of >0.2 in score was categorised as ‘improvement’; a change of<−0.2 was categorised as ‘deterioration’ of services; the rest was categorised as ‘no change’. The cut-offs followed the approach suggested in the WHO QI manual [8] to strike a balance between potential random variation and documenting actual improvement (or deterioration). We used SPSS, version 27 (SPSS Inc. Chicago, Illinois, USA) for all analyses. We analysed the changes in various domains of childcare from baseline to post-intervention with the Wilcoxon signed-rank test, considering a *P*-value of <0.05 as statistical significance.

## RESULTS

The baseline assessments in hospitals in Kyrgyzstan and Tajikistan defined many areas of care that required substantial improvement (Figure S1 and Figure S2 in the **Online Supplementary Document**). This was followed by two years of a multifaceted approach to improving QoC, which was implemented in the hospitals. Most hospitals in both countries achieved improvements across case management, hospital policies, and service organisation indicators, which were significant in some aspects of care.

### Clinical aspects of care

**Figure 1:**
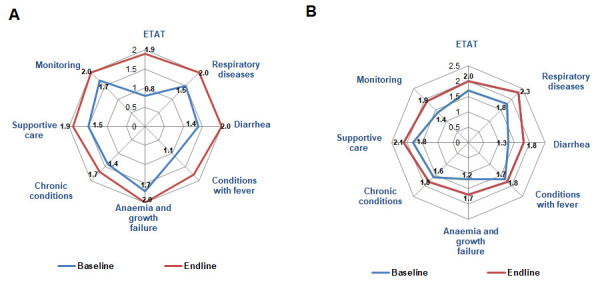
Comparison of changes in case management. **Panel A.** Tajikistan. **Panel B.** Kyrgyzstan.

**Figure 2:**
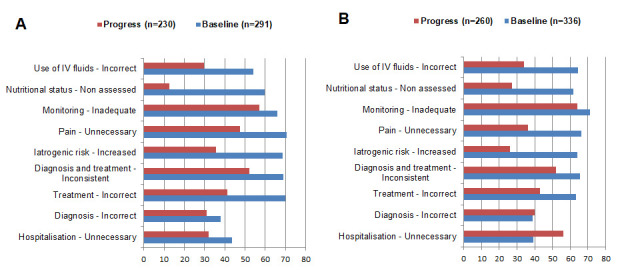
Review of patients’ charts (baseline and progress). **Panel A.** Tajikistan. **Panel B.** Kyrgyzstan.

The quality of triage and emergency care improved in Tajikistan (baseline x̄ = 0.8; endline x̄ = 1.9; *P* = 0.005), while the change was not significant in Kyrgyzstan (baseline x̄ = 1.7; endline x̄ = 2.0; *P* = 0.139) (**Figure 1**, Panels A and B). The management of pneumonia, including the use of appropriate antibiotics and oxygen therapy, improved in most hospitals in both countries. The score for assessment of suspected pneumonia increased in both Kyrgyzstan (baseline x̄ = 2.1; endline x̄ = 2.7; *P* = 0.018) and Tajikistan (baseline x̄ = 1.6; endline x̄ = 2.1; *P* = 0.020) (Figure S1 and Figure S2 in the **Online Supplementary Document**). Compliance with indications for hospitalisation for ARIs also improved in both Kyrgyzstan (baseline x̄ = 1.3; endline x̄ = 2.0; *P* = 0.034) and Tajikistan (baseline x̄ = 1.3; endline x̄ = 1.7; *P* = 0.034). The analysis of children’s care records also showed that the incorrect treatment of children with ARI decreased from 70% to 41% of cases in Tajikistan and from 63% to 43% in Kyrgyzstan (**Figure 2**).

Unnecessary, painful, and invasive procedures decreased in Tajikistan from 70% to 48% of cases and in Kyrgyzstan from 66% to 36% (**Figure 2**), indicating an area for further improvement. The iatrogenic risk in reviewed patient charts was considerably reduced in Tajikistan from 69% to 36%, and in Kyrgyzstan from 64% to 26%, due to the use of salbutamol, nebuliser therapy, and reduced intravenous infusions, especially in children with bronchial obstruction.

In more than half of the hospitals in both countries, the management of children with diarrhoea improved in Kyrgyzstan (baseline x̄ = 1.3; endline x̄ = 1.8; *P* = 0.028) and in Tajikistan (baseline x̄ = 1.4; endline x̄ = 2.0; *P* = 0.042). Children received oral zinc in only half of the cases, at the expense of the patients. Hospitals still did not provide zinc at the endline.

Monitoring and follow-up improved in Kyrgyzstan (baseline x̄ = 1.5; endline x̄ = 1.9; *P* = 0.021), but the change was not statistically significant in Tajikistan (baseline x̄ = 1.7; endline x̄ = 2.0; *P* = 0.123). The assessment of children's physical development improved significantly in both countries. Failure to assess development decreased in Tajikistan from 60% to 13% and in Kyrgyzstan from 62% to 27% of reviewed cases (**Figure 2**). Monitoring charts were not commonly implemented, and caregivers were insufficiently informed and not involved in the monitoring of the children.

Changes in management of children with chronic diseases were few, with a 0.2–0.3 scoring difference between the two assessments and with a x̄ score of 1.7 in Tajikistan and 1.8 in Kyrgyzstan (*P* = 0.175). Most hospital staff showed difficulties with the treatment of diabetes, asthma, and other chronic diseases in the presence of intercurrent diseases (diarrhoea or fever). Patients with anaemia or low weight commonly did not receive adequate nutrition, proper treatment, and monitoring.

The endline assessment found that irrational prescribing of antibiotics for ARI and diarrhoea, and the excessive use of drugs with unproven efficacy (especially in intensive care units), remained a problem (*e.g.* sedatives prescribed by neurologists due to overdiagnosis of intracranial hypertensive conditions) (Figure S1 and Figure S2 in the **Online Supplementary Document**).

Supportive care improved in Kyrgyzstan (baseline x̄ = 1.8; endline x̄ = 2.1; *P* = 0.021) and Tajikistan (baseline x̄ = 1.5; endline x̄ = 1.9; *P* = 0.009). Most hospitals in both countries still had a minimal budget to provide meals for children that were adequate in terms of frequency, size, and nutritional quality. The situation improved in five out of nine hospitals in Kyrgyzstan, but remained unchanged in Tajikistan, where free meals were unavailable in all hospitals. There was a trend toward improvement in nutrition counselling for children in both Kyrgyzstan (baseline x̄ = 1.4; endline x̄ = 1.8; *P =* 0.068) and Tajikistan (baseline x̄ = 0.5; endline x̄ = 1.8; *P* = 0.004), although the former change was non-significant in Kyrgyzstan.

The score for toy therapy improved significantly in Tajikistan (baseline x̄ = 0.4; endline x̄ = 1.5; *P* = 0.016) and had a trend for improvement in Kyrgyzstan (baseline x̄ = 0.8; endline x̄ = 1.5; *P* = 0.079). The review of children’s case records revealed that overall unjustified hospitalisation decreased in Tajikistan from 44% to 32% of cases, but increased in Kyrgyzstan from 40% to 56% of cases (**Figure 2**, Panels A and B) the analysis of case records showed that more than 45% of visits to the hospital admission rooms bypassed primary healthcare facilities. Criteria for hospitalisation of children with diarrhoea were not met in all hospitals with observed diarrhoea cases. Approximately 40% of all referred children could have been treated at the outpatient level.

### Organisational factors

**Figure 3:**
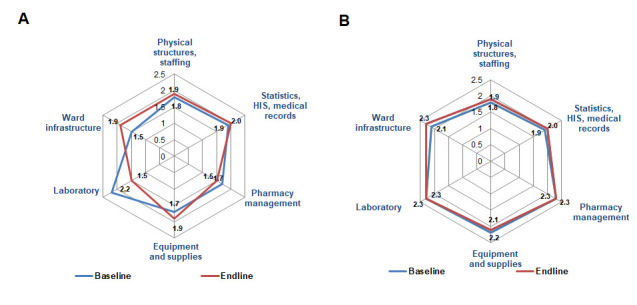
Comparison of changes in hospital support services. **Panel A.** Tajikistan. **Panel B.** Kyrgyzstan.

In both countries, the physical infrastructure, staffing, and basic services had positive but not statistically significant changes from 1.8 to 1.9 (*P* = 0.143) (**Figure 3**, Panels A and B). In both countries, almost all paediatric wards were not provided with cribs, resulting in mothers sharing a bed with their babies. Efforts made by hospital administrations led to improved infrastructure of the children's wards in most hospitals, with a trend of improvement observed in Kyrgyzstan (baseline x̄ = 2.1; endline x̄ = 2.3; *P* = 0.084) and an improvement in Tajikistan (baseline x̄ = 1.5; endline x̄ = 1.9; *P* = 0.022), particularly in sanitary and hygienic facilities, organisation of playrooms, and food preparation areas, where mothers could cook or warm up meals for children.

Equipment and supplies did not change in either Tajikistan (baseline x̄ = 1.7; endline x̄ = 1.9; *P* = 0.440) or Kyrgyzstan (baseline x̄ = 2.2; endline x̄ = 2.1; *P* = 0.863). Many hospitals in both countries have equipped their emergency units with essential drugs and equipment, providing diazepam for the treatment of seizures and offering systematic staff training in triage and emergency care, as documented at the endline.

The availability and use of equipment for oxygen therapy in children's wards improved. In Kyrgyzstan, the implementation of oxygen therapy for respiratory diseases initially had a higher rating and remained at a higher level (baseline x̄ = 2.1; endline x̄ = 2.4; *P* = 0.084), and in Tajikistan, the score was initially lower and increased at endline (baseline x̄ = 1.5; endline x̄ = 2.1; *P* = 0.043). Oxygen concentrators and pulse oximeters were observed in almost all hospitals of both countries during assessments and supervisory visits.

Staffing did not improve between the two assessments. There were not enough paediatric resuscitators, neonatologists, paediatricians, and infectious disease specialists. There was no staff rotation in the hospitals, and frequent on-call duties were common.

In both countries, the pharmaceutical management and supplies did not improve, remaining at 2.3 (*P* = 0.498) in Kyrgyzstan and decreasing from 1.7 to 1.5 (*P* = 0.341) in Tajikistan (**Figure 3**, Panels A and B). Supply with basic medicines (range and availability except in emergency units) was suboptimal in Tajikistan. Almost all hospitals in both countries were experiencing problems with insufficient numbers of basic equipment and consumables (*i.e.* electric suction devices, spacers, oxygen masks, child-sized catheters and nasogastric tubes, flowmeters, otoscopes).

In all hospitals, laboratory services were one of the weakest areas. These services maintained a score of 2.3 (*P* = 0.859) in Kyrgyzstan and worsened from 2.2 to 1.5 (*P* = 0.018) in Tajikistan. Free basic laboratory tests were limited to infants in Tajikistan and to those <6 years in Kyrgyzstan. Expensive tests, such as ferritin, C-reactive protein, procalcitonin, blood cultures, sensitivity to antibiotics, and hepatitis serology, were at the parents' expense.

### Systemic aspects of care

Overall compliance with children's rights improved in both countries. The endline assessment recorded a decrease in unnecessary, painful, and invasive procedures, including intravenous and intramuscular injections. In all hospitals, parents were allowed to stay with their children, and mothers could breastfeed their children on demand, including in the intensive care unit. Caretakers could prepare food for their children using their own products in designated facilities, which included a dining area. According to parent interviews, their awareness of sick child care issues improved due to better maternal education and informing parents on sick child and satisfaction with treatment and stay in hospitals increased. Respectful care was provided in 67–80% of hospitals in both countries, with an improvement in both Kyrgyzstan (baseline x̄ = 1.9; endline x̄ = 2.2; *P* = 0.024) and Tajikistan (baseline x̄ = 1.4; endline x̄ = 2.0; *P* = 0.007) (Figure S3, Panels A and B in the **Online Supplementary Document**).

Assessments of the results, along with the supervisor’s reports of staff competencies, indicated improvement in all project hospitals. Scores for audit and use of clinical guidelines improved in Tajikistan (baseline x̄ = 1.3; endline x̄ = 2.0; *P* = 0.005), and had a trend for improvement in Kyrgyzstan (baseline x̄ = 1.5; endline x̄ = 1.8; *P* = 0.086).

## DISCUSSION

The findings demonstrated substantial improvements in the quality of paediatric care across hospitals in Kyrgyzstan and Tajikistan over two years, following the implementation of a comprehensive QI approach. We framed the study within the European QoC framework [8], focussing on the key areas of case management, hospital policies, organisation of services, and supportive hospital services. The results highlight the potential of a structured, multifaceted intervention to improve healthcare quality. These activities were aligned with the development of the national governance in QoC and strengthened local QI committees. These committees were organised and sustained by a Ministry of Health order, building capacity in supervision and clinical guidelines, training, and participation in semi-annual QI review workshops. Activities also revealed ongoing challenges that will depend on sustained support and interest in the process from the government and, to some extent, the donor community for further improvements and their maintenance.

### Improvements in case management and clinical care

We observed the most notable improvements in case management, particularly in the management of ARI and emergency care. The quality of triage and emergency services improved in both Kyrgyzstan and Tajikistan. This improvement can probably be attributed to the intensive clinical training, practical sessions, on-the-job drills, and ongoing supportive supervision that were integrated into the intervention. The use of clinical guidelines, regular audits, and enhanced staff management might have facilitated adherence to best practices in the care of children with ARI and other common paediatric conditions. These changes align with the growing body of evidence on the importance of targeted training and regular supervision in improving clinical outcomes for children, particularly in low-resource settings [14–16].

One crucial positive outcome was the reduction in unnecessary and painful procedures, such as intramuscular injections and intravenous infusions. This indicates a shift away from practices rooted in the overmedicalisation of care, a persistent issue that can be traced back to the legacy of the Soviet healthcare system [3,17,18]. However, despite these advances, the irrational use of antibiotics and the overuse of medications with unproven efficacy persisted in both countries. This challenge reflects broader systemic issues, including the free sale of antibiotics in pharmacies, which exacerbates over-prescription and contributes to growing concerns about antibiotic resistance. Caretakers’ expectations, as well as diagnostic uncertainty, could contribute to overmedicalisation behaviours. Overcoming these ingrained practices will require not only continued educational efforts but also stronger regulatory oversight and changes in public health policies to restrict the inappropriate use of antibiotics. A detailed analysis of the irrational use of antibiotics is provided in the paper on paediatric care in Tajikistan (data not published).

### Chronic disease management

While improvements in the management of ARI and acute conditions were significant, the management of chronic diseases such as asthma, diabetes, and anaemia showed limited progress. This limited improvement may stem from the compartmentalised nature of healthcare in these countries, where care for chronic diseases is often siloed into specific departments. Such divisions complicate the management of comorbid conditions, especially when acute and chronic diseases coexist, as is often the case in children with conditions like pneumonia and malnutrition. Furthermore, chronic disease management in paediatric care requires long-term follow-up and monitoring, which remains a gap in the current system.

This finding underscores the need for a more holistic approach to child healthcare that integrates chronic disease management into the broader framework of paediatric care. The absence of a systematic approach to managing children with chronic diseases may hinder the effectiveness of treatment and long-term outcomes. Additionally, there is a need for greater emphasis on family-centred care, which involves parents in the management of chronic conditions.

### Infrastructure and equipment

We observed improvements in hospital infrastructure, including paediatric wards, playrooms, and food preparation areas, in many hospitals in both countries, especially in Tajikistan. However, these improvements were modest, and many hospitals still lacked basic equipment and supplies. In Kyrgyzstan, equipment shortages persisted, and the availability of basic medicines was suboptimal, particularly for non-emergency conditions. This limited availability of essential medicines and medical supplies is a significant barrier to providing high-quality care, especially for children with complex conditions or those in need of specialised treatments. The lack of child-sized medical equipment, such as paediatric oxygen masks and nasogastric tubes, further exacerbates the situation.

The disparities between the two countries suggest that Tajikistan has made more substantial progress in improving infrastructure, likely due to stronger support from hospital management, donors, and local initiatives to raise funds for improvements. The situation with basic medicines was better in Kyrgyzstan, requiring substantial measures in Tajikistan. Free laboratory tests are limited to infants in Tajikistan and children under 6 years in Kyrgyzstan. Both countries continue to struggle with limited budgets for healthcare and the absence of adequate resources for children's nutrition, medication, and follow-up care [6,7]. Structural improvements require sustained financial investment, political will, and local leadership to create a more supportive environment for paediatric care.

### Staffing and human resource constraints

While the intervention contributed to improvements in staff competencies through training and regular supervision, staffing shortages continue to be a critical challenge in both countries. The shortage of paediatric specialists, such as neonatologists and paediatric resuscitators, along with the lack of specialised training in chronic disease management, limits the capacity of hospitals to deliver high-quality care. In many hospitals, young doctors face low wages and a lack of incentives, contributing to high turnover and a scarcity of qualified personnel. Additionally, the ageing workforce and the migration of healthcare professionals to other countries further exacerbate staffing shortages. This staffing crisis highlights the need for comprehensive health workforce policies that address local recruitment, retention, and training needs.

The shortage of paediatric specialists and the lack of staff rotation often led to overwork and burnout, which can negatively impact the QoC. Furthermore, frequent staff turnover leads to gaps in knowledge and continuity of care, making it harder to implement and maintain QIs over time. Addressing these staffing issues requires investment in human resources, improved working conditions, and targeted retention strategies, such as offering competitive salaries and providing career development opportunities. Supportive supervision and collaborative reviews among peers that we implemented are also claimed to support staff, while other authors defined group problem solving, collaboration, and training as QI strategy component categories that had significant effects [19–21].

### Continuity of care and hospitalisation practices

A significant challenge identified in both countries is the continuity of care between primary healthcare (PHC) and hospitals. Many children were referred to hospitals without meeting the criteria for hospitalisation, a trend that continues to drive unnecessary admissions and place additional strain on hospital resources. Additionally, the high number of hospital visits that bypass PHC facilities points to a lack of trust in PHC services, especially in rural areas. This issue, combined with the absence of clear discharge instructions and follow-up care, requirements to fill ‘daybeds’ and financial considerations of hospitals, further complicates the patient journey and undermines the overall QoC.

Unjustified hospitalisation remains a significant problem in both countries [22], particularly for children with conditions that could be managed at the outpatient level. Hospital financing requirements and providers’ incentives, as well as parents’ pressing requests for hospitalisation, especially for children from vulnerable and poor families, were seen as the drivers for unnecessary hospitalisations in Kyrgyzstan. Addressing this issue requires strengthening the PHC system, improving the QoC at the first point of contact, and fostering trust between caregivers and healthcare providers [3,6,7,23]. Additionally, addressing current hospital financing constraints and ensuring better communication between hospitals and PHC providers through shared medical records and comprehensive discharge planning is essential to improving continuity of care and reducing unnecessary hospitalisations.

### Strengths and limitations of the study

The main strength of our study was a standardised assessment methodology with validated tools applied in both countries at the baseline and endline surveys. We enhanced the objectivity and impartiality of the assessment process by involving external international experts alongside trained national assessors, as well as by validating peer-review assessment and analysis processes in both countries. The relatively small sample of hospitals, the absence of a control group, and the non-independence of the local assessors were the limitations of our study. We attempted to overcome this by involving international experts and adopting a standardised approach. Without a control group, we cannot be certain that the improvements were due to the interventions, but as no other major projects took place at the same time, they are the most plausible explanation.

## CONCLUSIONS

We demonstrated that significant improvements in emergency care, triage, and pneumonia management were possible in both countries, alongside a reduction in the use of unnecessary and invasive procedures. Irrational antibiotic prescribing, overmedicalisation, and unjustified hospitalisation continue to be issues, which might require systemic changes at the national level supported by both political commitment and community engagement. The management of chronic conditions showed a modest improvement. A more integrated care approach is needed to address these challenges. Infrastructure improvements were more noticeable in Tajikistan, while both countries still faced issues like inadequate supplies, equipment shortages, limited access to laboratory tests, and staffing shortages. Building clinical competencies in hospital staff and providing supportive supervision should be sustained by persistently addressing systemic barriers, such as ineffective financial policies, staffing issues, and the lack of free essential medicines, laboratory tests, and meals for hospitalised children. Continuity of care between primary healthcare and hospitals remains weak, leading to unnecessary hospitalisations and poor follow-up care, which will require strengthening the PHC system’s gatekeeper role.

Overall, the QI interventions led to improvements in case management and hospital policies; however, gaps in infrastructure, chronic disease management, and staffing still required attention. We recommend scaling up good practices built in the project hospitals to other hospitals. This action across the country will require an integrated approach, including policies, capacity building, improved training curricula, a system of supportive supervision, regular joint peer reviews, and an active role for hospital QI committees and professional associations.

## Additional Material


Online Supplementary Document
