# Supplementary material for: Paediatric care in hospitals in Kyrgyzstan and Tajikistan: impact of a quality improvement initiative
Source: J Glob Health. 2025 Oct 24;15:04272. doi: 10.7189/jogh.15.04272 (PMC12548769; doi:10.7189/jogh.15.04272)

Supplement to: Babayeva B, Ospanova Z, Aminov O, Shukurova V, Tilenbaeva N, Yusupova S, Avaliani A, Weber MW, Jullien S, Kuttumuratova A; Kyrgyzstan-Tajikistan Quality of Hospital Care for Mothers and Children study group. Paediatric care in hospitals in Kyrgyzstan and Tajikistan: impact of a quality improvement initiative. J Glob Health. 2025;15:04272.

Figure 1S. Heat chart of hospital paediatric care in Kyrgyzstan (baseline and endline assessments)

Hospital coding		1			2			3			4			5			6			7			8			9			Summary		
**Hospital Support Services**		Baselin	Progress	Differenc	Baselin	Progres	Differenc	Baselin	Progress	Differenc	Baselin	Progress	Differenc	Baselin	Progress	Differenc	Baselin	Progress	Difference	Baselin	Progress	Differenc	Baselin	Progress	Difference	Baselin	Progres	Differenc	**Baseli**	**Progres**	**Difference**
1,1	Physical structures, staffing and	1,5	1,8	0,3	1,5	2,1	0,6	1,3	1,0	-0,3	2,6	2,7	0,1	2,0	2,1	0,1	2,1	2,2	0,1	1,3	1,4	0,1	2,0	2,0	0,0	1,8	1,8	0,0	1,8	1,9	0,1
1,2	Statistics, health management information systems and medical records	3,0	2,2	-0,8	1,5	2,0	0,5	2,2	1,4	-0,8	1,7	2,1	0,4	1,9	2,1	0,2	1,9	2,0	0,1	1,8	2,0	0,2	2,0	2,2	0,2	1,2	1,8	0,6	1,9	2,0	0,1
1,3	Pharmacy management and me	2,5	1,9	-0,6	2,5	2,0	-0,5	2,0	2,0	0,0	2,8	2,8	0,0	3,0	2,7	-0,3	2,8	2,6	-0,2	2,0	2,7	0,7	1,4	2,1	0,7	1,5	2,3	0,8	2,3	2,3	0,1
1,4	Equipment and supplies	2,2	1,3	-0,9	2,0	2,0	0,0	2,3	1,0	-1,3	2,5	2,7	0,2	2,3	2,5	0,2	2,3	2,5	0,2	2,0	2,0	0,0	1,8	2,3	0,5	2,3	2,5	0,2	2,2	2,1	-0,1
1,5	Diagnostic services: laboratory	2,0	1,8	-0,2	2,5	2,0	-0,5	2,6	2,0	-0,6	2,5	2,6	0,1	3,0	2,6	-0,4	3,0	2,8	-0,2	1,8	2,3	0,5	2,0	2,4	0,4	1,5	2,3	0,8	2,3	2,3	0,0
1,6	Ward infrastructure	2,0	2,1	0,1	1,5	2,1	0,6	2,0	1,5	-0,5	2,4	2,5	0,1	2,3	2,5	0,2	2,6	2,8	0,2	2,0	2,4	0,4	2,0	2,4	0,4	2,0	2,1	0,1	2,1	2,3	0,2
**Case management**																															
2	Emergency triage and treatmen	1,4	1,8	0,4	2,5	1,7	-0,8	1,2	1,0	-0,21	1,5	2,2	0,7	1,7	2,0	0,3	1,9	2,0	0,1	1,6	2,0	0,4	1,7	2,1	0,4	2,0	2,9	0,9	1,7	2,0	0,2
2.1.	A system of triage is in place	1,0	2,0	1,0	2,0	1,5	-0,5	0,8	1,2	0,5	1,8	2,0	0,3	1,5	2,0	0,5	1,0	2,0	1,0	1,0	2,0	1,0	1,0	1,6	0,6	1,5	3,0	1,5	1,3	1,9	0,6
2.2.	Appropriate management of ai	1,0	2,0	1,0	1,5	1,5	0,0	1,5	1,0	-0,5	1,5	2,0	0,5	1,5	2,0	0,5	2,0	2,0	0,0	1,0	2,5	1,5	2,0	2,0	0,0	2,0	3,0	1,0	1,6	2,0	0,4
2.3.	Appropriate management of br	1,0	1,7	0,7	2,5	1,7	-0,8	1,0	1,0	0,0	2,5	2,5	0,0	1,5	2,0	0,5	1,5	2,0	0,5	2,0	2,0	0,0	2,0	2,6	0,6	2,0	3,0	1,0	1,8	2,1	0,3
2.4.	Appropriate management of sh	1,0	2,0	1,0	2,0	1,5	-0,5	1,6	1,0	-0,6	2,0	2,0	0,0	2,0	2,0	0,0	2,0	2,0	0,0	2,0	2,0	0,0	2,0	1,7	-0,3	2,0	3,0	1,0	1,8	1,9	0,1
2.5.	Appropriate management of co	1,0	1,6	0,6	2,2	1,5	-0,7	1,5	1,0	-0,5	1,5	2,3	0,8	2,0	2,0	0,0	2,0	2,0	0,0	2,0	2,0	0,0	2,0	2,6	0,6	2,5	3,0	0,5	1,9	2,0	0,1
2.6.	Management of severe trauma	2,0	2,6	0,6	1,0	1,5	0,5	1,3	1,0	-0,3	0,0	2,0	2,0		2,0			2,0			2,0						3,0				
2.7.	Timely treatment	0,0	1,5	1,5	2,0	2,5	0,5	1,0	1,0	0,0	2,0	2,0	0,0	2,0	2,0	0,0	3,0	2,0	-1,0	1,0	2,0	1,0	1,0	1,8	0,8	2,0	3,0	1,0	1,6	2,0	0,4
2.8.	Admission criteria	2,0	1,5	-0,5	2,0	1,5	-0,5	1,0	1,0	0,0	1,0	3,0	2,0	1,5	2,0	0,5	2,0	2,0	0,0	2,0	2,0	0,0	2,0	2,0	0,0	2,0	2,0	0,0	1,7	1,9	0,2
3	Case management of respirator	2,1	2,2	0,1	2,5	2,7	0,2	1,5	1,8	0,4	1,8	2,7	1,0	1,6	2,2	0,6	1,9	2,2	0,4	2,0	2,1	0,1	1,5	2,3	0,8	1,8	2,0	0,2	1,8	2,3	0,4
3.1.	Admission criteria	1,0	2,0	1,0	1,0	2,0	1,0	1,0	1,0	0,0	1,0	3,0	2,0	1,0	2,0	1,0	2,0	2,0	0,0	2,0	2,0	0,0	1,0	2,0	1,0	2,0	2,0	0,0	1,3	2,0	0,7
3.2.	Clinical assessment for suspecte	1,3	2,0	0,7	2,0	2,8	0,8	1,3	1,7	0,4	2,0	2,3	0,3	1,0	1,6	0,6	1,5	2,3	0,8	2,0	2,0	0,0	2,0	2,0	0,0	1,3	1,6	0,3	1,6	2,0	0,4
3.3.	Investigations for suspected pn	2,0	3,0	1,0	2,5	3,0	0,5	1,5	2,0	0,5	1,5	3,0	1,5	2,0	2,5	0,5	2,0	3,0	1,0	3,0	2,5	-0,5	2,0	3,0	1,0	2,0	2,5	0,5	2,1	2,7	0,7
3.4.	Administration of appropriate a	1,7	2,5	0,8	2,6	2,5	-0,1	1,5	1,7	0,2	2,0	2,5	0,5	1,5	2,0	0,5	1,5	2,0	0,5	2,0	1,7	-0,3	1,0	2,2	1,2	0,8	1,6	0,8	1,6	2,1	0,5
3.5.	Oxygen therapy	2,3	2,0	-0,3	2,6	2,8	0,2	1,3	1,8	0,5	3,0	2,7	-0,3	2,0	2,6	0,6	2,0	2,3	0,3	2,0	2,5	0,5	2,0	2,2	0,2	2,0	2,8	0,8	2,1	2,4	0,3
3.6.	Complications of pneumonia	2,0	2,0	0,0	2,5	3,0	0,5	1,4	2,0	0,6	2,0	3,0	1,0	2,0	2,0	0,0	2,0	2,0	0,0		2,0		2,0	3,0	1,0	2,0	2,0	0,0	2,0	2,3	0,3
3.7.	Management of children with b	2,8	3,0	0,3	2,0	2,8	0,8	2,0	2,7	0,7	2,0	3,0	1,0	1,5	2,5	1,0	1,5	2,3	0,8	2,0	2,2	0,2	1,0	2,7	1,7	2,3	2,1	-0,2	1,9	2,6	0,7
3.8.	Management of children with la	1,5	2,0	0,5	2,5	3,0	0,5	2,0	2,0	0,0	2,0	2,7	0,7	1,5	2,5	1,0	2,0	2,5	0,5		2,5		2,0	2,0	0,0	2,5	1,5	-1,0	1,8	2,3	0,5
3.9.	Management of children with l	2,0	2,3	0,3	2,0	2,3	0,3	1,0	1,5	0,5	1,0	2,0	1,0	1,5	2,0	0,5	2,0	2,0	0,0		1,5		1,0	2,0	1,0		2,0		1,5	2,0	0,5
3.10.	Discharge	2,0	3,0	1,0	2,0	3,0	1,0	1,0	1,3	0,3	1,0	3,0	2,0	1,6	2,5	1,0	2,0	1,6	-0,4	1,0	2,0	1,0	1,0	2,0	1,0	1,7	2,0	0,3	1,5	2,3	0,8
4	Case management of diarrhea	1,0	1,7	0,7	1,0	1,5	0,5				1,4			1,3	1,5	0,2	1,7	1,8	0,1	1,5	2,0	0,5	1,4			1,0	2,1	1,1	1,3	1,8	0,5
4.1.	Admission criteria	1,0	1,0	0,0	1,0	1,0	0,0				2,0			2,0	1,5	-0,5	1,5	2,0	0,5	2,0	2,0	0,0	2,0			1,0	1,5	0,5	1,5	1,6	0,1
4.2.	Assessment of degydration	3,0	2,0	-1,0	1,0	1,0	0,0				1,0			1,0	1,5	0,5	1,5	2,0	0,5	1,0	2,0	1,0	1,0			0,5	2,0	1,5	1,3	1,6	0,3
4.3.	Rehydration plan	1,3	2,0	0,7	1,0	1,4	0,4				1,0			1,0	1,4	0,4	1,5	1,0	-0,5	1,0	2,0	1,0	1,0			1,2	2,2	1,0	1,1	1,7	0,5
4.4.	Use of antibiotics and other dru	1,0	2,0	1,0	1,0	2,0	1,0				1,0			1,0	1,5	0,5	2,0	3,0	1,0	2,0	2,0	0,0	2,0			0,7	2,3	1,6	1,3	2,1	0,8
4.5.	Feeding	1,0	1,6	0,6	1,0	1,6	0,6				2,0			2,0	1,8	-0,2	2,0	1,5	-0,5	1,0	2,0	1,0	1,0			1,4	2,4	1,0	1,4	1,7	0,2
4.6.	Discharge	1,7	1,7	0,0	1,0	1,7	0,7				1,5			1,0	1,0	0,0	1,5	1,0	-0,5	2,0	2,0	0,0				1,3	2,0	0,7	1,4	1,5	0,1
5	Case management of other con	2,3	1,5	-0,8	1,5	1,5	0,0	1,4	1,8	0,4	1,8	1,7	-0,1	1,4	1,8	0,4	1,7	1,4	-0,3	2,0	2,1	0,1	2,0	2,3	0,3	1,1	1,7	0,6	1,7	1,8	0,1
5.1.	Admission criteria	3,0	2,0	-1,0	1,5	1,0	-0,5	1,0	2,0	1,0	1,5	2,0	0,5	2,0	3,0	1,0	1,5	2,0	0,5	2,0	2,0	0,0	2,0	3,0	1,0	1,0	1,5	0,5	1,7	2,1	0,3
5.2.	Differential diagnosis and inves	1,5	1,5	0,0	1,5	1,5	0,0	1,4	1,5	0,2	1,5	2,5	1,0	1,0	2,5	1,5	1,5	2,0	0,5	2,0	2,0	0,0	2,0	2,0	0,0	1,0	1,5	0,5	1,5	1,9	0,4
5.3.	Diagnosis and management of r	1,5	2,0	0,5	2,0	2,0	0,0	1,7			1,5			0,0	1,5	1,5	1,5	2,0	0,5		2,6			2,0			2,0				
5.4.	Diagnosis and management of u	1,5	1,7	0,2	1,5	1,8	0,3	1,4	1,5	0,1	2,0	1,2	-0,8	1,5	1,0	-0,5	2,0	1,5	-0,5	3,0	2,2	-0,8	2,0	2,5	0,5	1,3	2,0	0,7	1,8	1,7	-0,1
5.5.	Diagnosis and management of a	1,5	2,0	0,5	2,0	1,5	-0,5	1,5	1,8	0,3	2,0	1,3	-0,7	1,0	1,0	0,0	1,5	1,0	-0,5	1,0	2,0	1,0	2,0	2,2	0,2	1,0	1,0	0,0	1,5	1,5	0,0
5.6.	Assessment and management c	1,5	2,0	0,5	1,0	2,0	1,0	1,5	2,0	0,5	2,0			2,0	2,0	0,0	2,0	1,0	-1,0		2,0		2,0	2,0	0,0	1,0	2,0	1,0	1,6	1,9	0,3
5.7.	Rheumatic fever										1,5			1,5	2,0	0,5	1,5	2,0	0,5					2,0							
5.8.	Fever lasting longer than 7 days	1,0	1,5	0,5		1,0					2,0			2,0	2,3	0,3	2,0	2,0	0,0		2,0			2,4							
6	Case management of anaemia a	0,8	1,1	0,3	1,0	1,7	0,7	0,1	1,5	1,4	1,3	2,2	1,0	1,3	1,5	0,3	1,4	1,6	0,23	1,3	1,9	0,6	1,8	2,0	0,3	1,4	1,6	0,2	1,1	1,7	0,5
6.1.	Differential diagnosis and inves	0,0	1,0	1,0	2,0	2,0	0,0	0,0	1,0	1,0	1,0	2,3	1,3	1,0	1,0	0,0	1,0	1,0	0,0	2,0	1,5	-0,5	2,0	2,0	0,0	1,0	1,5	0,5	1,1	1,5	0,4
6.2.	Management of anaemia	0,0	1,5	1,5	0,3	1,9	1,6	0,0	1,5	1,5	1,0	2,0	1,0	1,0	2,0	1,0	1,0	1,0	0,0	1,0	1,5	0,5	2,0	2,0	0,0	2,0	1,8	-0,3	0,9	1,7	0,8
6.3.	Identification of children with growth failure (acute or chronic malnutrition) and differential diagnosis	0,0	1,0	1,0	0,8	1,4	0,7	0,5	1,6	1,1	2,0	2,8	0,8	1,5	2,0	0,5	2,0	2,0	0,0	1,0	2,0	1,0	2,0	1,6	-0,4	0,8	1,7	0,9	1,2	1,8	0,6
6.4.	Management of children with growth failure (acute or chronic malnutrition)	2,0	1,0	-1,0	0,0	1,5	1,5	0,0	1,7	1,7	1,0	2,0	1,0	1,5	2,0	0,5	1,5	2,0	0,5		2,5		1,0	2,5	1,5	1,8	1,5	-0,3			
7	Case management of chronic co	2,0	1,7	-0,3	1,0	1,7	0,7				1,6	2,0	0,4	1,5	1,6	0,1	1,9			2,0	1,9	-0,1	2,0	2,1	0,1	1,5	1,8	0,3	1,7	1,8	0,15
7.1.	Type I diabetes	1,2	1,0	-0,2		1,7	1,7				1,5			1,0	1,7	0,7	1,5			2,0	2,0	0,0	2,0	2,3	0,3	1,5	2,0	0,5	1,2	1,6	
7.2.	Celiac disease										2,0			2,0			2,0							2,0							
7.3.	Asthma	1,5	1,6	0,1		2,0					1,0	2,0	1,0	1,0	1,5	0,5	1,5	2,0	0,5	2,0	1,6	-0,4	2,0	2,2	0,2	1,5	1,3	-0,2	1,2	1,6	0,4
7.4.	Cystic fibrosis					1,3					1,0			1,0			1,5							2,0							
7.5.	HIV/ AIDS										2,5			2,5	1,0	-1,5	2,5														
7.6.	Leukaemia										2,0			2,0			2,5							2,0							
7.7.	Development disorders	1,0	1,5	0,5	1,0	1,8	0,8				1,0			1,0			1,5				2,0			2,3			2,0				
8	Supportive care	1,9	2,0	0,1	1,1	2,0	0,9	1,5	1,4	-0,1	2,1	2,3	0,2	1,9	2,1	0,23	2,3	2,3	0,0	1,8	2,4	0,7	1,5	2,2	0,7	1,8	2,2	0,4	1,8	2,1	0,3
8.1.	Promotion of breastfeeding	3,0	2,2	-0,8	1,5	2,0	0,5	1,2	1,8	0,6	2,5	3,0	0,5	2,5	3,0	0,5	3,0	2,7	-0,3	2,0	2,0	0,0	1,0	2,8	1,8	1,8	2,6	0,8	2,1	2,5	0,4
8.2.	Nutritional management of the	1,0	2,0	1,0	1,0	1,6	0,6	1,2	1,4	0,2	2,0	2,0	0,0	2,0	1,6	-0,4	2,0	1,6	-0,4	1,0	2,2	1,2	1,0	2,0	1,0	1,0	1,8	0,8	1,4	1,8	0,4
8.3.	Use of intravenous fluids	1,3	2,0	0,7	1,0	2,0	1,0	1,0	2,0	1,0	1,5	1,5	0,0	1,5	1,0	-0,5	2,0	1,0	-1,0	2,0	2,3	0,3	1,0	2,0	1,0	1,3	1,8	0,5	1,4	1,7	0,3
8.4.	Management of fever	3,0	2,5	-0,5	2,0	2,0	0,0	2,0	2,0	0,0	2,0	3,0	1,0	2,0	3,0	1,0	2,0	3,0	1,0	3,0	3,0	0,0	2,0	2,5	0,5	3,0	2,0	-1,0	2,3	2,6	0,22
8.5.	Management of pain	2,3	2,7	0,4	1,5	2,0	0,5	1,6	1,7	0,1	2,0	2,0	0,0	2,0	2,0	0,0	2,0	2,0	0,0				2,0			1,7	1,8	0,1	1,7	1,6	-0,1
8.6.	Blood transfusion	2,5	3,0	0,5	2,0	2,0	0,0	3,0	2,0	-1,0	3,0	3,0	0,0	3,0	1,0	-2,0	3,0	3,0	0,0	2,0	2,6	0,6	2,0	2,0	0,0	2,6	2,7	0,1	2,6	2,4	-0,2
8.7.	Oxygen therapy	2,8	2,0	-0,8	1,0	2,0	1,0	2,0	1,8	-0,2	3,0	2,8	-0,2	2,0	2,6	0,6	1,5	2,3	0,8	2,0	2,8	0,8	2,0	2,6	0,6	2,3	2,6	0,3	2,1	2,4	0,3
8.8.	Toys and play therapy	0,0	0,0	0,0	0,0	1,3	1,3	0,0	0,7	0,7	1,0	2,0	1,0	0,0	3,0	3,0	2,5	3,0	0,5	2,0	2,3	0,3	1,0	1,3	0,3	1,0	0,0	-1,0	0,8	1,5	0,7
9	Monitoring and follow up	1,0	2,3	1,3	1,0	2,0	1,0	1,2	1,8	0,6	2,0	2,4	0,4	1,5	1,8	0,3	1,8	1,7	-0,1	1,3	1,7	0,4	1,8	1,8	0,0	1,5	1,6	0,1	1,5	1,9	0,4
**Policies and organization of services**																															
10	Infection prevention	2,0	1,7	-0,3	1,5	2,0	0,5	2,5	1,7	-0,8	1,9	2,7	0,8	1,8	2,6	0,9	2,5	2,5	0,0	2,0	2,4	0,4	1,6	2,6	1,0	1,9	2,6	0,7	2,0	2,3	0,4
10,1	Infection control policies	2,0	2,0	0,0	2,0	2,2	0,2	2,7	2,0	-0,7	1,5	3,0	1,5	1,0	2,4	1,4	2,0	2,2	0,2	1,0	2,2	1,2	2,0	3,0	1,0	2,0	2,4	0,4	1,8	2,4	0,6
10,2	Hand washing	2,0	2,0	0,0	2,0	2,3	0,3	1,6	2,0	0,4	1,5	2,0	0,5	2,0	1,6	-0,4	2,0	2,3	0,3	2,0	3,0	1,0	1,0	3,0	2,0	2,0	3,0	1,0	1,8	2,4	0,6
10,3	Use of gloves	2,0	2,0	0,0	2,0	2,2	0,2	2,4	2,0	-0,4	3,0	3,0	0,0	2,0	3,0	1,0	3,0	2,8	-0,2	3,0	3,0	0,0	1,0	3,0	2,0	2,0	3,0	1,0	2,3	2,7	0,4
10,4	Patients' and staff personal hyg	2,0	1,0	-1,0	1,5	2,0	0,5	1,0	1,0	0,0	0,0	3,0	3,0	1,0	2,0	1,0	2,0	2,0	0,0	3,0	1,0	-2,0	2,0	2,0	0,0	3,0	2,0	-1,0	1,7	1,8	0,1
10,5	Isolation	1,0	1,0	0,0	1,5	2,0	0,5	3,0	1,0	-2,0	0,0	1,5	1,5	0,0	3,0	3,0	2,0	3,0	1,0	1,0	1,0	0,0	1,0	1,0	0,0	2,0	1,3	-0,8	1,3	1,6	0,4
10,6	Laundry	2,0	2,0	0,0	1,5	2,0	0,5	3,0	2,0	-1,0	3,0	3,0	0,0	2,0	3,0	1,0	3,0	2,6	-0,4	2,0	3,0	1,0	2,0	3,0	1,0	2,0	3,0	1,0	2,3	2,6	0,3
10,7	Management of wastes	2,0	2,0	0,0	1,5	2,0	0,5	3,0	2,0	-1,0	3,0	3,0	0,0	3,0	3,0	0,0	3,0	3,0	0,0	2,0	3,0	1,0	2,0	3,0	1,0	1,0	2,8	1,8	2,3	2,6	0,4
10,8	Sterilization	2,0	2,0	0,0	2,0	2,0	0,0	3,0	2,0	-1,0	3,0	3,0	0,0	3,0	3,0	0,0	3,0	3,0	0,0	2,0	3,0	1,0	2,0	3,0	1,0	1,0	3,0	2,0	2,3	2,7	0,3
11	Guidelines and audit	1,1	1,4	0,3	1,0	1,8	0,8	1,9	1,1	-0,8	1,8	2,2	0,4	1,9	2,1	0,23	2,0	2,1	0,1	1,3	1,9	0,6	1,6	2,3	0,7	1,0	1,3	0,3	1,5	1,8	0,3
11,1	Guidelines are available	1,0	1,0	0,0	1,0	2,0	1,0	3,0	1,0	-2,0	3,0	1,8	-1,2	3,0	2,0	-1,0	2,5	2,3	-0,2	2,0	2,0	0,0	2,0	2,0	0,0	1,0	2,0	1,0	2,1	1,8	-0,3
11,2	Guidelines are used	1,0	2,0	1,0	1,0	2,0	1,0	2,8	1,0	-1,8	1,0	1,5	0,5	1,0	2,0	1,0	2,0	2,0	0,0	1,0	2,0	1,0	1,0	2,3	1,3	1,0	1,5	0,5	1,3	1,8	0,5
11,3	Learning resources are availabl	1,0	1,2	0,2	1,0	2,2	1,2	3,0	1,2	-1,8	2,5	2,0	-0,5	1,0	2,0	1,0	2,0	2,2	0,2	1,0	2,0	1,0	3,0	2,3	-0,8	1,0	2,0	1,0	1,7	1,9	0,2
11,4	In service training occurs	1,0	1,2	0,2	1,0	1,7	0,7	1,5	1,0	-0,5	1,5	2,5	1,0	2,0	2,0	0,0	1,5	2,0	0,5	1,0	2,2	1,2	1,0	2,3	1,3	1,0	1,7	0,7	1,3	1,8	0,6
11.5.	Continuous professional educat	1,0	1,3	0,3	1,0	1,6	0,6	0,7	1,0	0,3	2,0	2,3	0,3	1,0	2,0	1,0	2,0	3,0	1,0	1,0	1,0	0,0	2,0	3,0	1,0	1,0	1,5	0,5	1,3	1,9	0,6
11.6.	Team working is encouraged	1,0	1,6	0,6	1,5	1,6	0,1	0,7	2,0	1,3	1,5	2,6	1,1	2,0	3,0	1,0	2,0	2,0	0,0	2,0	2,0	0,0	1,0	3,0	2,0	1,0	2,0	1,0	1,4	2,2	0,8
11.7.	Audit and review process is in p	1,0	2,0	1,0	1,5	2,0	0,5	1,5	1,0	-0,5	1,5	3,0	1,5	3,0	2,0	-1,0	2,0	2,0	0,0		2,0		1,0	1,8	0,8	1,0	2,0	1,0			
11.8.	Audit and case reviews are con	1,8	1,8	0,0	1,0	2,0	1,0	1,8	1,3	-0,5	1,5	2,2	0,7	2,0	2,1	0,1	2,0	2,0	0,0		2,0		2,0	2,3	0,3	1,0	1,7	0,7			
12	Access to hospital care and cont	2,0	2,0	0,0	2,0	2,0	0,0	2,0	1,1	-0,9	1,7	2,3	0,6	1,8	2,0	0,2	2,1	2,2	0,1	2,0	2,3	0,3	2,0	2,5	0,5	2,0	2,2	0,2	2,0	2,1	0,1
13	Respectful care	2,0	1,8	-0,2	2,0	2,3	0,3	1,5	1,4	-0,1	2,1	2,6	0,5	1,8	2,3	0,5	2,3	2,5	0,2	2,2	2,6	0,4	1,5	2,1	0,6	1,5	2,0	0,5	1,9	2,2	0,3

Figure 2S. Heat chart of hospital paediatric care in Tajikistan (baseline and endline assessments)

Hospital coding		1			2			3			4			5			6			7			8			9			10			Summary		
		Baselin	Progress	Differenc	Baselin	Progress	Differenc	Baselin	Progress	Differenc	Baselin	Progress	Differenc	Baselin	Progress	Differenc	Baselin	Progress	Differenc	Baselin	Progress	Differenc	Baselin	Progress	Differenc	Baselin	Progress	Differenc	Baselin	Progress	Differenc	Baselin	Progress	Difference
**Section 1 - Hospital Support Services**																																		
1.1.	Structure of medical units, staffing and basic services	2,3	1,8	-0,5	2,1	2,1	0,0	1,8	2	0,2	2	2,3	0,3	1,8	2,7	0,9	1,3	1,7	0,4	2,2	1,8	-0,4	1,5	1,6	0,1	1,5	2,1	0,6	1	1	0,0	1,8	1,9	0,2
1.2.	information systems and medical records	3	2,4	-0,6	3	2	-1,0	2,8	2	-0,8	2	2,5	0,5	1,8	2,2	0,4	1	1,6	0,6	1,3	2	0,7	1	1,6	0,6	1	2,1	1,1	1,6	1,6	0,0	1,9	2,0	0,2
1.3.	Provision of drugs	2,4	1	-1,4	3	1,5	-1,5	2,6	1,7	-0,9	1,4	1,8	0,4	0,5	1,4	0,9	1,6	1,1	-0,5	1,5	1,6	0,1	1,5	1,6	0,1	1,5	1,4	-0,1	1,4	1,4	0,0	1,7	1,5	-0,3
1.4.	Medical equipment and consumab	2,8	2,2	-0,6	1	1,6	0,6	3	2	-1,0	2	2,2	0,2	1	1,8	0,8	1,3	1,3	0,0	2	2	0,0	1,2	1,6	0,4	1,2	2	0,8	1,7	1,8	0,1	1,7	1,9	0,1
1.5.	Diagnostic services: laboratory	2,9	1	-1,9	3	1	-2,0	2,9	1,7	-1,2	1,7	1,7	0,0	1,8	1,8	0,0	1,7	1	-0,7	1,7	1,6	-0,1	2,2	1,7	-0,5	2,2	1,8	-0,4	1,5	1,5	0,0	2,2	1,5	-0,7
1.6.	Infrastructure of the pediatric dep	2,5	2,4	-0,1	1,8	1,9	0,1	1,7	2,1	0,4	1,2	1,8	0,6	1,5	2,4	0,9	1,1	1,5	0,4	1,5	1,3	-0,2	1,1	1,9	0,8	1,1	2,2	1,1	1,7	1,9	0,2	1,5	1,9	0,4
**Section 2 - Management of patients with**																																		
2	Conducting medical triage and em	1	2,2	1,2	0	1,8	1,8	0	1,8	1,8	1,1	2,3	1,2	0,9	1,8	0,9	0,5	1,6	1,1	1,4	2,3	0,9	1,1	1,5	0,4	1,1	1,8	0,7	0,9	1,4	0,5	0,8	1,9	1,1
2,1	Conducting on-site medical triage	1	2	1,0	0	2	2,0	0	1	1,0	0,8	2,4	1,6	1	1,7	0,7	0	1	1,0	1,5	2,5	1,0	1	2	1,0	1	2	1,0		1		0,7	1,8	1,1
2,2	Airway patency	1	2,2	1,2	0	2	2,0	0	2	2,0	1	2,4	1,4	1	1,7	0,7	0	2	2,0	1,5	2,4	0,9	1	1	0,0	1,5	2	0,5	1	1,1	0,1	0,8	1,9	1,1
2,3	Labored breathing	1	2,3	1,3	0	2	2,0	0	2	2,0	1	2,4	1,4	1	1,7	0,7	0,5	2	1,5	1,5	2,4	0,9	1	2	1,0	1,5	1	-0,5	1	1,5	0,5	0,9	1,9	1,1
2,4	Shock	1	2,2	1,2	0	2	2,0	0	2	2,0	1	2	1,0	1	1,7	0,7	1	2	1,0	1	2,4	1,4	1	1	0,0	2	2	0,0	1	1	0,0	0,9	1,8	0,9
2,5	Convulsions	1	2,5	1,5	0	2	2,0	0	2	2,0	0,8	2,4	1,6	0,25	1,9	1,7	1	1,9	0,9	1,5	2,4	0,9	1,5	1	-0,5	1	2	1,0	1	1,4	0,4	0,8	2,0	1,1
2,6	Serious injuries and wounds		2,3		0	1	1,0		2	2,0					2			1	1,0		2	2,0		2	2,0		2		1					
2,7	Speed of care provision		2		0	2	2	0	2	2	1	2,4	1,4	1	1,7	0,7	0	2	2	2	2,2	0,2	1,5	1	-0,5	1,5	2	0,5	1	1,8	0,8	0,9	1,9	1,0
2,8	Admission criteria	0	2	2,0	0	1,5	1,5	0	2	2,0	2	2	0,0	1	1,8	0,8	1	1	0,0	1	2	1,0	1	2	1,0	0	2	2,0	1	2	1,0	0,7	1,8	1,1
3	*Management of children with resp*	2	2,2	0,2	1,7	1,9	0,2	2	1,7	-0,3	2	2,3	0,3	1,3	1,8	0,5	1	1,9	0,9	1,5	2,4	0,9	1	1,6	0,6	1	2	1,0	1	2,1	1,1	1,5	2,0	0,5
3,1	Admission Criteria	1	2	1,0	2	2	0,0	1,5	2	0,5	2	2	0,0	1	1	0,0	1	1	0,0	1	2	1,0	1	1	0,0	1	2	1,0	1	2	1,0	1,3	1,7	0,5
3,2	Clinical evaluation for suspected p	1,6	2,2	0,6	2	2	0,0	2	1	-1,0	1,7	2	0,3	1	2	1,0	1	2	1,0	1,5	2,2	0,7	1,5	2	0,5	1	2	1,0	1	2	1,0	1,4	1,9	0,5
3,3	Examination for suspected pneum	2	2,2	0,2	2	2	0,0	2	2	0,0	3	2,8	-0,2	1,5	2	0,5	1	2	1,0	2	2,4	0,4	1,5	2	0,5	0	1	1,0	1	2,3	1,3	1,6	2,1	0,5
3,4	Prescribing appropriate antibiotics	1,5	2,1	0,6	2	1,8	-0,2	3	1	-2,0	1,5	2,4	0,9	0,5	1,7	1,2	1	2	1,0	1	2,2	1,2	1	2	1,0	1	2,2	1,2	1	2	1,0	1,4	1,9	0,6
3,5	Oxygen therapy	2,2	2,3	0,1	1	2,1	1,1	2	2	0	2	2	0	2	2	0	0	2,5	2,5	2	2,2	0,2	0,5	2	1,5	2	2	0		2		1,5	2,1	0,6
3,6	Complications of pneumonia	2	2	0,0	2	1,8	-0,2		2		2	2,3	0,3	2	2	0,0	1	1,5	0,5	2	2,2	0,2	1,5	2	0,5	2	2,5	0,5	2	2,5	0,5	1,8	2,1	0,2
3,7	Management of children with bror	2,3	2,3	0,0	1	2	1,0	2	1	-1,0	1,8	2,3	0,5	0,75	1,9	1,2	1	2	1,0	1	2,5	1,5	0,5	1	0,5	0	2,2	2,2	2	2,4	0,4	1,2	2,0	0,7
3,8	Treatment of children with laryng	2	2,1	0,1	1	1,8	0,8	2	2	0,0	2	2,3	0,3	1	1,9	0,9	1	2	1,0	1,5	2,5	1,0	1	2	1,0	1	2	1,0		2		1,4	2,1	0,7
3,9	Treatment of children with long-te	3	2	-1,0	2	1,5	-0,5		2		2	2	0,0		1,9	1,9	2	2	0,0	2	2,2	0,2	1	1	0,0	1	2	1,0		2		1,6	1,9	0,2
3.10.	Discharge	2	2,2	0,2	2	2,1	0,1	2	2	0,0	2	2,4	0,4	1	1,9	0,9	1	2,4	1,4	1	2	1,0	1	1	0,0	1	2,4	1,4	1	2	1,0	1,4	2,0	0,6
4	*Management of children with diarr*	1	2,1	1,1		1,7			1,8		2,3	2,4	0,1	2,3	1,5	-0,8	0,3	1,7	1,4	1	2,2	1,2	1	2	1,0	1	2,2	1,2	2,3	2,3	0,0	1,4	2,0	0,6
4,1	Admission criteria	2	2	0		1			2		3	2,5	-0,5	2	1	-1	1	1	0	1	2	1	1	2	1	0	2,2	2,2		2,5		1,4	1,8	0,4
4,2	Assessing the degree of dehydrati	1	2	1,0		2			2		3	2,5	-0,5	2,5	2	-0,5	1	2	1,0	1	2,5	1,5	1	2	1,0	1	2,5	1,5		2,5		1,5	2,2	0,7
4,3	Compliance with rehydration plan	1,8	2	0,2		2			2		3	2,5	-0,5	1,8	1	-0,8	0	2	2,0	1	2,4	1,4	1	2	1,0	1	2	1,0		2,5		1,4	2,0	0,7
4,4	Use of antibiotics and other drugs	1,6	2	0,4		2			2		2	2,5	0,5	1,6	1	-0,6	0	2	2,0	1	2	1,0	1	2	1,0	1	2	1,0		2		1,2	2,0	0,8
4,5	Diet	1,6	2	0,4		1			2		1	2	1,0	2,8	2	-0,8	0	1	1,0	1	2,2	1,2	1	2	1,0	2	2	0,0		2		1,3	1,8	0,5
4,6	Discharge	1,3	2,3	1,0		2			1		2	2,4	0,4	2	2	0,0	0	2,5	2,5	1	2	1,0	1	2	1,0	1	2,5	1,5		2		1,2	2,1	0,9
5	*Management of children with feve*	1,2	2,1	0,9	0,6	1,8	1,2	1	1,8	0,8	1,7	2,1	0,4	1	1,6	0,6	0,8	1,5	0,7	1,7	2,2	0,5	1,3	1,5	0,2	1,3	2	0,7	0,8	1,7	0,9	1,1	1,8	0,7
5,1	Admission criteria	1	2	1,0	1	2	1,0	1	2	1,0	2	2	0,0	1	2	1,0	1	1	0,0	1,5	2,5	1,0	1	1	0,0	1	2,3	1,3	0	1	1,0	1,1	1,8	0,7
5,2	Differential diagnosis and examina	0	1,8	1,8	1	2	1,0	1	2	1,0	2	2	0,0	1	1	0,0	1	2	1,0	1,5	2,4	0,9	1	2	1,0	1	2,5	1,5	1	2	1,0	1,1	2,0	0,9
5,3	Diagnosis and treatment of meningitis		2,4		0	2	2,0		2		1,6	2	0,4		1		1	1	0,0	2	2,2	0,2	1,5	2	0,5	1	2	1,0		1		1,2	1,8	0,6
5,4	Diagnosis and treatment of urinary	2	2,5	0,5	0	2	2,0		1		2	2,5	0,5	1	2	1,0	1	2	1,0	2	2	0,0	1,5	1	-0,5	1	2	1,0	1	2	1,0	1,3	1,9	0,6
5,5	Diagnosis and treatment of acute c	2	2	0,0	1	1	0,0		2		0,5	1,5	1,0	1	1	0,0	0	1	1,0	1,5	2	0,5	1,5	2	0,5	1,5	1	-0,5	1	2	1,0	1,1	1,6	0,4
5,6	Diagnosis and treatment of other s	1	2,4	1,4	1	2	1		2		2	2,4	0,4		2		1	2	1	2	2,2	0,2	1	2	1	1,5	2	0,5		2		1,4	2,1	0,7
5,7	Rheumatic fever		2		0	1	1,0		2		2	2,4	0,4		2		0	1	1,0	1,5	2	0,5	1	1	0,0	1	2	1,0		2		0,9	1,7	0,8
5,8	Prolonged fever (more than 7 days)		2		0	2	2,0		2		2	2	0,0		2		1	2	1,0	1,5	2	0,5	1,5	1	-0,5	2	2,4	0,4		2		1,3	1,9	0,6
6	*Management of patients with anei*	2,2	2,3	0,1	2,2	2	-0,2	2	1,5	-0,5	2,1	2,4	0,3	1	1,8	0,8	1,8	2	0,2	1,1	2,3	1,2	1,6	1,5	-0,1	1,6	2,2	0,6	1	2	1,0	1,7	2,0	0,3
6,1	Differential diagnosis and examina	2	2,5	0,5	2	2	0,0	2	1	-1,0	1,3	2	0,7	0	1	1,0	2	2	0,0	1	2	1,0	1	1	0,0	2	2	0,0	1	1	0,0	1,4	1,7	0,2
6,2	Treatment	2	2,5	0,5	2	2	0,0	2	1	-1,0	2	2	0,0	1	2	1,0	1	2	1,0	1,5	2	0,5	1,5	1	-0,5	1	2	1,0	1	2	1,0	1,5	1,9	0,4
6,3	Identification of children with malnutrition and differential diagnosis	1,5	2	0,5	2	2	0,0	2	2	0,0	3	2,8	-0,2	1,2	2	0,8	2	2	0,0	1	2,5	1,5	2	2	0,0	1,5	2,5	1,0		2		1,8	2,2	0,4
6,4	Management of children with malnutrition (acute and chronic malnutrition)	2,5	2	-0,5	2,5	2	-0,5	2	2	0	2	2,8	0,8	2	2,2	0,2	2	2	0	1	2,5	1,5	2	2	0	2	2,4	0,4		2		2,0	2,2	0,2
7	*Management of patients with chro*	1,6	2,1	0,5		1,8		2,3	1,5	-0,8	1,1	1,8	0,7	2	1,6	-0,4	0,6	1,3	0,7	1,5	1,8	0,3	1,2	1,1	-0,1	1,2	1,8	0,6	0,9	1,7	0,8	1,4	1,7	0,3
7,1	Diabetes mellitus type I		2,2			2		2	1	-1,0	1	2	1,0		2		1	1	0,0	1,5	1,5	0,0	1	1	0,0	2	2	0,0		2		1,4	1,7	0,3
7,2	Celiac disease		2			1,5			1		0	1	1,0		1	1,0	0	1	1,0	2	2	0,0	1	1	0,0	0	2	2,0		1				
7,3	Bronchial asthma	1	2	1,0		2		2	2	0,0	1	2	1,0	2	2	0,0	1	1	0,0	1,5	1,5	0,0	1,5	1	-0,5	2	1	-1,0		1		1,5	1,7	0,2
7,4	Cystic fibrosis	1	1,5	0,5		1,5			1		0	1	1,0		1	1,0	0	1	1,0	1,5	1,5	0,0	1,5	1	-0,5	0	2	2,0		1		0,6	1,3	0,7
7,5	HIV AIDS		2,1			2			2		2	2	0,0		2		1	2	1,0	2	2	0,0	1,5	1	-0,5	2	2	0,0		2				
7,6	Leukemia		2,3			1,5			2		2	2,3	0,3	2	2	0	1	1	0	1	2	1	1	1	0	0,5	2	1,5		2		1,3	1,8	0,6
7,7	Developmental delay	3	2,4	-0,6		2		1,5	1,5	0,0	2	2	0,0		1	1,0	0	2	2,0	1	2	1,0	2	2	0,0	2	2	0,0		1		1,4	1,8	0,4
8	Supportive care	1,8	1,9	0,1	1,8	2,1	0,3	2	1,9	-0,1	2	2,1	0,1	1,4	1,9	0,5	1	1,5	0,5	1,4	2,1	0,7	1,2	1,9	0,7	1,2	2,1	0,9	0,9	1,9	1,0	1,5	1,9	0,5
8,1	Breastfeeding support	3	2	-1,0	3	2,1	-0,9	2	2	0,0	3	2,5	-0,5	3	2,5	-0,5	2	2	0,0	2	2,5	0,5	2	2	0,0	2	2,2	0,2	2	2	0,0	2,4	2,2	-0,2
8,2	Diet therapy for a sick child	1	2	1,0	1	2,1	1,1	0	2	2,0	0,2	1	0,8	0	1	1,0	0	2	2,0	0	2	2,0	1	2	1,0	1	2	1,0	1	2	1,0	0,5	1,8	1,3
8,3	Infusion therapy	1	2	1,0	1	2	1,0	2	2	0,0	3	2,5	-0,5	2	2	0,0	2	1	-1,0	1,5	2,2	0,7	1,5	2	0,5	1	2	1,0	1	2	1,0	1,6	2,0	0,4
8,4	Management of patients with feve	1	2	1,0	1	1,8	0,8	3	2	-1,0	2	2,3	0,3	2	2	0,0	1	2	1,0	1	2	1,0	1	2	1,0	1	2	1,0	1	1,5	0,5	1,4	2,0	0,6
8,5	Management of patients with pain	3	1,5	-1,5	3	1,8	-1,2	3	1	-2	2	2,3	0,3		2	2	0	1	1	1,5	2,4	0,9	1,5	1	-0,5	1	2	1	1	2	1	1,8	1,7	-0,1
8,6	Blood transfusion	3	2,3	-0,7	3	2,5	-0,5	2	2	0,0	3	2,8	-0,2	2	2	0,0	1,7	2,5	0,8	3	2,4	-0,6	3	2	-1,0	1,5	2,5	1,0		2		2,5	2,3	-0,2
8,7	Oxygen therapy	2	2,3	0,3	1	2,5	1,5	2	2	0,0	2,6	2,6	0,0	0,8	1,6	0,8	1,2	2,5	1,3	2	2	0,0	0,5	2	1,5	2	2	0,0	0	1,6	1,6	1,4	2,1	0,7
8,8	Toys and play therapy	1	1	0,0	1	2,1	1,1	2	2	0,0	0	1	1,0	0	2	2,0	0	0	0,0	0	1	1,0	0	2	2,0	0	2	2,0	0	2	2,0	0,4	1,5	1,1
9	Monitoring and observation of pat	2	1,8	-0,2	2	2	0,0	2,6	1,8	-0,8	2,3	2,5	0,2	1,4	2	0,6	1,3	2	0,7	1,2	2,2	1,0	1,2	1,8	0,6	1,2	1,9	0,7	1,3	1,8	0,5	1,7	2,0	0,3
**Section 3 - Hospital Policies and Organiza**																																		
10	Prevention of nosocomial infectio	1	2,3	1,3	1,5	2,3	0,8	1,8	2	0,2	1,5	2	0,5	2,3	2,6	0,3	2,6	2,1	-0,5	1,7	1,8	0,1	1,6	1,9	0,3	1,6	1,9	0,3	1,1	1,9	0,8	1,7	2,1	0,4
10,1	Nosocomial infection control	2	2,3	0,3	2	2,2	0,2	2	2	0,0	2,4	2	-0,4	2,5	2	-0,5	3	2	-1,0	1	2,4	1,4	1	2	1,0	1	2,2	1,2	0	2	2,0	1,7	2,1	0,4
10,2	Hand washing	2	2,5	0,5	2	2,5	0,5	2	2	0,0	3	3	0,0	3	3	0,0	3	2,5	-0,5	2	2,4	0,4	1	1	0,0	1	2	1,0	1	2	1,0	2,0	2,3	0,3
10,3	Using gloves	1,8	2,5	0,7	1	2,5	1,5	2	2	0,0	1,5	2,3	0,8	2,6	2,6	0,0	2,2	2,5	0,3	2	2,2	0,2	2	2	0,0	2	2	0,0	2	2	0,0	1,9	2,3	0,4
10,4	Personal hygiene of patients and r	2	2	0,0	1	2,4	1,4	1	2	1,0	1	2	1,0	2	2	0,0	3	2	-1,0	1	1,5	0,5	1	2	1,0	1	1	0,0	2	2	0,0	1,5	1,9	0,4
10,5	Isolation of infectious patients	2			1	1	0,0	1	2	1,0	0	1	1,0	0	2	2,0	2	0	-2,0	1	0	-1,0	1	2	1,0	0	1	1,0	0	1	1,0	0,8	1,1	0,3
10,6	Bed sheet	2,0	2	0,0	1,0	2,4	1,4	2,0	2	0,0	1,0	2	1,0	2,7	2,8	0,1	3,0	2,5	-0,5	2,0	2,2	0,2	2,0	2	0,0	2,0	2,5	0,5	1,0	1,8	0,8	1,9	2,2	0,4
10,7	Waste disposal	2,0	2,4	0,4	2,0	2,5	0,5	2,0	2	0,0	2,0	2	0,0	3,0	3	0,0	3,0	2,5	-0,5	2,0	2,2	0,2	2,0	2	0,0	3,0	3	0,0	2,0	2	0,0	2,3	2,4	0,1
10,8	Sterilization	2,4	2,4	0,0	2,0	2,5	0,5	2,0	2	0,0	1,6	2	0,4	3,0	3	0,0	2,0	2,5	0,5	2,5	2,2	-0,3	2,5	2	-0,5	3,0	2	-1,0	2,0	2	0,0	2,3	2,3	0,0
11	Management, staff training and au	1,4	2,3	0,9	1,1	2	0,9	1,5	2	0,5	1,9	2,2	0,3	1,3	2	0,7	1,1	2	0,9	1,3	1,8	0,5	1,1	1,8	0,7	1,1	2,1	1,0	1,0	1,9	0,9	1,3	2,0	0,7
11,1	Availability of manuals	2,0	2,8	0,8	2,0	2,1	0,1	2,0	2	0,0	2,0	2	0,0	1,0	2	1,0	1,0	2	1,0	1,0	2	1,0	1,5	2	0,5	1,0	2	1,0	2,0	2	0,0	1,6	2,1	0,5
11,2	Using of guidelines	1,7	2,5	0,8	1,0	2,4	1,4	1,8	2	0,2	1,0	2,3	1,3	0,5	2	1,5	1,0	2	1,0	1,0	2	1,0	1,0	2	1,0	1,0	2,4	1,4	1,0	2	1,0	1,1	2,2	1,1
11,3	Availability of learning resources	0,5	2,4	1,9	1,0	2	1,0	1,0	2	1,0	1,5	2	0,5	0,0	1	1,0	0,0	2	2,0	2,0	1	-1,0	1,5	2	0,5	1,0	2	1,0	0,0	1		0,9	1,7	0,9
11,4	Ensuring timely staff training	1,2	2,4	1,2	1,0	2	1,0	1,0	2	1,0	1,0	1,5	0,5	1,0	1,8	0,8	1,5	2	0,5	1,0	2	1,0	1,0	2	1,0	2,0	2	0,0	1,0	2	1,0	1,2	2,0	0,8
11,5	Providing continuing professional	1,0	2	1,0	1,0	2	1,0	1,0	2	1,0	0,7	1,9	1,2	2,0	2,3	0,3	2,0	2	0,0	1,0	2	1,0	1,0	2	1,0	1,0	2	1,0	1,0	2	1,0	1,2	2,0	0,9
11,6	Encouraging teamwork	1,2	2	0,8	1,0	2	1,0	2,0	2	0,0	3,0	3	0,0	2,0	2,5	0,5	2,0	2	0,0	1,5	2	0,5	1,0	2	1,0	1,0	2,2	1,2	1,0	2	1,0	1,6	2,2	0,6
11,7	Audit	2,0	2	0,0	1,0	1,8	0,8	1,0	2	1,0	2,0	2,5	0,5	2,0	2,5	0,5	1,0	2	1,0	1,5	2	0,5	1,0	1	0,0	1,0	2,4	1,4	1,0	2	1,0	1,4	2,0	0,7
11,8	Audit and case analysis	1,7	2	0,3	1,0	1,8	0,8	1,8	2	0,2	1,9	2,5	0,6	2,0	2,5	0,5	0,9	2	1,1	1,5	2	0,5	1,0	1	0,0	1,0	2	1,0	1,0	2	1,0	1,4	2,0	0,6
12	Availability of hospital care	2,8	2	-0,8	1,7	2	0,3	1,4	1,8	0,4	2,2	2,2	0,0	1,9	2,2	0,3	0,9	1,8	0,9	1,7	1,8	0,1	1,8	2	0,2	1,8	2,2	0,4	1,0	2,4	1,4	1,7	2,0	0,3
13	Respect for children's rights	1,8	2	0,2	1,4	1,6	0,2	1,3	1,9	0,6	1,9	2,5	0,6	1,8	2,5	0,7	1,5	1,5	0,0	0,9	1,6	0,7	1,1	1,7	0,6	1,1	2,2	1,1	0,9	2,4	1,5	1,4	2,0	0,6

Figure 3S. Comparison of changes in policies and organisation of services. **Panel A.** Tajikistan. **Panel B.** Kyrgyzstan.

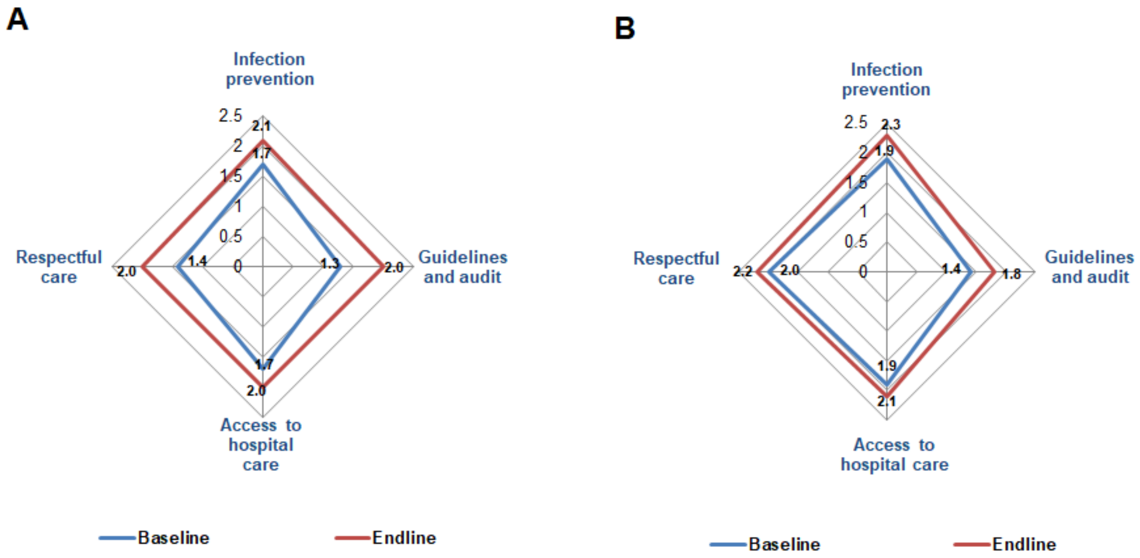

Supplement: Online Supplementary Document [file jogh-15-04272-s001.pdf]
